# Teste de Exercício Cardiopulmonar em Pacientes Pós-COVID-19: Novos Insights sobre a Intolerância ao Exercício

**DOI:** 10.36660/abc.20230058

**Published:** 2023-03-06

**Authors:** Marina Petersen Saadi, Anderson Donelli da Silveira

**Affiliations:** 1 Hospital de Clínicas de Porto Alegre Porto Alegre RS Brasil Serviço de Cardiologia Hospital de Clínicas de Porto Alegre, Porto Alegre, RS – Brasil; 2 Programa de Pós-Graduação em Cardiologia: Ciências Cardiovasculares Universidade Federal do Rio Grande do Sul Porto Alegre RS Brasil Programa de Pós-Graduação em Cardiologia: Ciências Cardiovasculares da Universidade Federal do Rio Grande do Sul, Porto Alegre, RS – Brasil; 3 Hospital Moinhos de Vento Porto Alegre RS Brasil Hospital Moinhos de Vento, Porto Alegre, RS – Brasil

**Keywords:** Pós Covid-19/complicações, Pneumopatias, Pandemia, Síndrome do Desconforto Respiratório do Adulto/reabilitação, Espirometria/métodos, Teste de Esforço, Medidas de Volume Pulmonar, Atividade Física

COVID-19 afetou milhares de pessoas em todo o mundo, e uma porcentagem considerável deles se queixa de limitação persistente ao exercício, sem relação com a gravidade da doença e persistindo mesmo meses após a infecção.^1 , 2^ Sabe-se que a forma grave da COVID-19 envolve predominantemente o sistema respiratório, apresentando síndrome do desconforto respiratório agudo. Pode-se especular que a limitação funcional persistente observada após a infecção pode ter origem no sistema respiratório, fato reforçado por estudos que mostraram limitação ventilatória na espirometria após a alta hospitalar.^3 , 4^

O teste cardiopulmonar de exercício (TECP) permite uma avaliação completa do sistema cardiorrespiratório. É o exame padrão-ouro para definir o prognóstico em diversas doenças cardíacas e pulmonares e pode discriminar os mecanismos subjacentes associados à redução da capacidade funcional.^5 , 6^

Nos Arquivos Brasileiros de Cardiologia, Milani et al.^7^ avaliaram 144 pacientes sem doença cardiovascular ou pulmonar prévia, com sintomas persistentes após a infecção por COVID, em média 11,5 semanas após a infecção, que foram comparados com 144 controles pareados adultos saudáveis. A maioria dos pacientes (92%) teve o teste do TCPE limitado pela fadiga muscular periférica, com mínima limitação pulmonar (6%) e cardiovascular (2%). A frequência e a magnitude da redução da capacidade funcional foram relacionadas à gravidade da doença, com 56% dos pacientes com COVID grave apresentando redução da capacidade funcional com menor VO_2_% em relação aos grupos menos graves. O VO_2_ nos limiares ventilatórios também foi reduzido nos indivíduos mais doentes.^7^

Os autores também fizeram uma abordagem diferente para uma análise de subgrupo em que pacientes que haviam se submetido a TCPE antes da covid por outro motivo tiveram testes pré e pós-COVID comparados. No grupo covid moderado, houve redução de 0,4 km/h na velocidade máxima e pequeno aumento no percentual de FC máxima prevista. No grupo moderado/grave, houve maior redução da velocidade máxima (1 mk/h) e redução significativa do VO_2_ pico (7,4%), provocando redução da capacidade física nos mesmos pacientes após COVID.^7^

O principal mecanismo de limitação funcional após COVID parece ter etiologia muscular periférica, embora respiração disfuncional e insuficiência cronotrópica também foram descritas como possíveis causas.^8^A redução da capacidade funcional por fadiga muscular decorrente da extração anormal de oxigênio na periferia pelo catabolismo muscular não parece estar relacionada apenas a períodos de repouso e isolamento e sim à própria doença COVID, fato verificado por estudos que mostraram redução no VO_2_ de pico associado, extração pobre de O_2_ periférico com índice cardíaco normal.^9^

Diante desses achados, é provável que a reabilitação cardiopulmonar com exercícios físicos prescritos e executados por profissionais qualificados possa melhorar os sintomas e as medidas objetivas da capacidade funcional. No entanto, são necessários ensaios clínicos randomizados com exercícios ou reabilitação cardiopulmonar.^10^
