# Supplementary material for: Teste de Exercício Cardiopulmonar em Pacientes Pós-COVID-19: Novos Insights sobre a Intolerância ao Exercício
Source: Arq Bras Cardiol. 2023 Mar 6;120(2):e20230058. [Article in Portuguese] doi: 10.36660/abc.20230058 (PMC10263460; doi:10.36660/abc.20230058)
Supplement: Translation

# Translation: Cardiopulmonary Exercise Testing in Post-COVID-19 Patients: New Insights Into the Exercise Intolerance

**Authors:** Marina Petersen Saadi, Anderson Donelli da Silveira

**Affiliations:** 1 Brazil Serviço de Cardiologia Hospital de Clínicas de Porto Alegre, Porto Alegre, RS – Brazil; 2 Brazil Programa de Pós-Graduação em Cardiologia: Ciências Cardiovasculares da Universidade Federal do Rio Grande do Sul, Porto Alegre, RS – Brazil; 3 Brazil Hospital Moinhos de Vento, Porto Alegre, RS – Brazil

**Keywords:** Post-Covid-19/complications, Lung Diseases, Pandemics, Respiratory Distress Syndrome Adult/rehabilitation, Spirometry/methods, Exercise Test, Lung Volume Measurements, Physical Activity

COVID-19 has affected thousands of people worldwide, and a considerable percentage of those complain of persistent exercise limitation, unrelated to the disease`s severity and persisting even months after the infection.^1 , 2^It is well known that the severe form of COVID-19 predominantly involves the respiratory system, presenting with acute respiratory distress syndrome. One could speculate that the persistent functional limitation seen after infection may originate in the respiratory system, a fact reinforced by studies that showed ventilatory limitation in spirometry after hospital discharge.^3 , 4^

Cardiopulmonary Exercise Test (CPET) allows a complete assessment of the cardiorespiratory system. It is the gold standard exam to define prognosis in several heart and lung diseases and can discriminate the underlying mechanisms associated with reduced functional capacity.^5 , 6^

In this *Arquivos Brasileiros de Cardiologia* , Milani et al.^7^evaluated 144 patients without a prior cardiovascular or pulmonary disease, with persistent symptoms after COVID infection, on average 11.5 weeks after infection, who were compared with 144 healthy adult matched controls. Most of the patients (92%) had the CPET test limited by peripheral muscle fatigue, with minimal pulmonary (6%) and cardiovascular (2%) limitation. The frequency and magnitude of the reduction in functional capacity were related to the severity of the disease, with 56% of patients with severe COVID presenting a reduction in functional capacity with a lower VO_2_% compared to the less severe groups. The VO_2_at ventilatory thresholds was also reduced in the sickest individuals.^7^

The authors also made a different approach for a subgroup analysis where patients who had undergone CPET before covid for another reason had pre- and post-COVID tests compared. In the moderate covid group, there was a reduction of 0.4 km/h in the maximum speed and a small increase in the percentage of predicted maximum HR. In the moderate/severe group, there was a greater reduction in maximum speed (1 mk/h) and a significant reduction in peak VO_2_(7.4%), eliciting a reduction in physical capacity in the same patients after COVID.^7^

The main mechanism of functional limitation after COVID seems to have a peripheral muscular etiology, although dysfunctional breathing and chronotropic insufficiency have also been described as possible causes.^8^The reduction in functional capacity because of muscle fatigue due to abnormal extraction of oxygen in the periphery by muscle catabolism does not seem to be related only to periods of rest and isolation but to the COVID disease itself, a fact verified by studies that showed a reduction in associated peak VO_2_poor peripheral O_2_extraction with normal cardiac index.^9^

Because of these findings, it is likely that cardiopulmonary rehabilitation with physical exercises prescribed and executed by qualified professionals could improve symptoms and objective measures of functional capacity. However, randomized clinical trials with exercise or cardiopulmonary rehabilitation are needed.^10^
